# The influence of social dominance orientation and right-wing authoritarianism on environmentalism: A five-year cross-lagged analysis

**DOI:** 10.1371/journal.pone.0219067

**Published:** 2019-07-10

**Authors:** Samantha K. Stanley, Taciano L. Milfont, Marc S. Wilson, Chris G. Sibley

**Affiliations:** 1 School of Psychology, Victoria University of Wellington, Wellington, New Zealand; 2 Discipline of Psychology, University of Canberra, Canberra, Australia; 3 School of Psychology, The University of Auckland, Auckland, New Zealand; Valparaiso University, UNITED STATES

## Abstract

Social dominance orientation (SDO) and right-wing authoritarianism (RWA) are ideological attitudes that predict lower concern for the environment and less willingness to act on climate change. Research generally shows that SDO and RWA exhibit moderate, negative relationships with environmentalism. We examine the longitudinal influence of SDO and RWA on people’s willingness to change their behaviour to benefit the environment in a national probability sample over five years. We show that both ideological attitudes relate to lower environmentalism across time and that the SDO effect was stronger than the RWA effect, yet the association from environmentalism to later endorsement of SDO is stronger than the reverse. Interestingly, these findings suggest that the more likely temporal association flows from environmentalism to SDO.

## Introduction

Research on the psychological foundations of climate change denial have highlighted the important role of two ideological attitudes: Social dominance orientation (SDO) and right-wing authoritarianism (RWA). People who endorse SDO want society to be structured hierarchically, with some groups at the top dominating over ‘lower status’ social groups, who are relegated to the bottom of the social hierarchy [1]. Right-wing authoritarians are those who prefer to conform to group norms and the orders of authority figures, while punishing those who do not conform [2]. SDO and RWA were initially developed to help explain attitudes towards social groups, with both Social Dominants and Authoritarians showing a preference for groups of people similar to themselves, and hostility towards those who are different [3].

However, the ideological attitudes broaden to predict attitudes towards the environment. Specifically, Social Dominants are more likely to oppose environmental policies [4], support environmental exploitation [5, 6], and deny climate change and its human causes [7–11]. Similarly, Authoritarians are less concerned about the environment [12], perceive climate change as less of a risk [13], and support punishing environmental activists—and not polluters [14].

While the research to date demonstrates the importance of SDO and RWA in understanding people’s attitudes and actions towards the environment, the extant literature has predominantly relied on cross-sectional analyses to examine this association. In one exception to this, Stanley, Wilson and Milfont [15] examined the associations between endorsement of RWA, SDO and environmentalism over time using a student sample surveyed twice over five months. They found that RWA, and *not* SDO, predicts an increase in climate change denial, while neither variable is related to changes in pro-environmental attitudes over time. We expand on Stanley et al.’s study by using a large, national probability sample spanning five years to examine how these variables relate over time in the general population.

### The association between SDO, RWA and environmentalism

Duckitt’s [3] dual-process model proposes that SDO and RWA are separate but interrelated, and together predict attitudes towards different social groups. The model has been widely used to understand intergroup relations, and shows the pathways from an individuals’ personality characteristics and worldviews to SDO and RWA and, in turn, favourability toward the ingroup and hostility toward outgroups. On the surface, SDO appears to be of interest exclusively to research on intergroup relations and, indeed, items in the SDO scale exclusively frame hierarchies based on intergroup relations.

However, Pratto et al. [4] present possibly the first analysis of the relationship between SDO and attitudes towards issues that are not solely focused on social groups, including attitudes towards environmental policies. They predicted that SDO would correlate with opposition to any policy that reduces inequality between humans and other species. Indeed, they found modest negative correlations between SDO and support for environmental policy. This is perhaps the first hint that for those endorsing SDO, nature might be another ‘outgroup’ to assert dominance over.

This idea of human dominance over nature is evident in anthropocentric depictions of our relationship with the natural environment, a belief that is at the core of the dominant social paradigm (DSP) [16]. The DSP indexes endorsement of ideological systems favouring growth and prosperity. Individuals invested in the DSP position themselves to dominate and exploit the environment, and are unmoved by appeals to protect resources [17]. Milfont et al. [10] argue that Social Dominants’ propensity for environmental exploitation exists “because SDO promotes human hierarchical dominance over nature” (p. 1127).

While a preference for dominance over nature could underlie Social Dominants’ environmental attitudes, there is also research to suggest the SDO–environmentalism link can instead be explained by support for using natural resources as a means to gain dominance over others. Specifically, Milfont and Sibley’s [5] hierarchy enforcement hypothesis of environmental exploitation claims that Social Dominants will support environmental exploitation only when this serves to enhance the gap between dominant and subordinate social groups. They showed that SDO predicts support for an environmentally hazardous mine only when the social elite gain disproportionate access to the resources this produces. Jackson et al. [6] similarly demonstrated that SDO predicts support for a mining operation unconditional of the environmental impact—but only when the ingroup (in this case, their own country) is set to gain (Study 2). Additionally, Social Dominants are happy to position a manufacturing plant that poses a serious risk to the environment in economically disadvantaged areas (Study 3). Therefore, acceptance of environmental exploitation may be motivated by the desire to dominate over other social groups by making use of nature, rather than the desire to dominate over the natural world itself.

Irrespective of *why* the relationship exists, a growing number of studies have provided evidence for the association between SDO and environmentalism, which paint a somewhat dismal picture for climate change mitigation. Social Dominants are less likely to believe in climate change and its human causes [7–10], prompting researchers to urge caution not to portray environmental action as threatening to the social hierarchy [7]. Milfont et al. [10] showed that Social Dominants are less concerned about the environment (Study 1), while also more accepting of exploitation of natural resources (Study 3). Moreover, greater average country-level SDO is associated with poorer environmental performance (Study 2). Social Dominants are more supportive of making use of natural resources, and hold generally more negative attitudes towards the environment [18]. Across nations, the SDO-environmentalism link is small but robust, with the association strengthened in areas with greater social inequality [11].

Alongside this research on SDO, Peterson, Doty and Winter [14] similarly found that RWA was related to statements that conceptualise environmental issues as overstated, environmental action as detrimental to the country, and environmentalists as deserving of punishment. Hoffarth and Hodson [19] similarly showed that Authoritarians view environmentalists as posing a threat to society, tradition, and the economy. Furthermore, and perhaps consistent with their denial of climate change [15, 19], Authoritarians are less likely to assume responsibility for acting on global warming, or intention to mitigate the problem [20].

Schultz and Stone [12] showed that RWA and environmentalism evidence a strong, negative relationship. Furthermore, they found both RWA and environmentalism explained unique variance in support for a controversial power plant. They interpret this association as due to the authoritarian preference for economic growth, with natural resources proving a useful avenue for income. Whilst an interest in conservation would prohibit excessive use of natural resources, Authoritarians prioritize growth and therefore dismiss environmental concerns. However, Reese [21] found that the dimensions of RWA differentially predict pro-environmental attitudes: authoritarian submission predicts greater pro-environmentalism, authoritarian aggression predicts lower pro-environmentalism, whilst the preference for tradition is unrelated.

Conclusions about why the relationships exist between these ideological attitudes and environmentalism (such as that it is motivated by a desire for dominance over nature, or to use nature to get ahead in the case of SDO, or the preference for tradition or authoritarian aggression for RWA) imply a directional model where one’s level of SDO or RWA influences environmental attitudes. Whilst this implied direction is consistent with theoretical predictions of how ideology relates to attitudes over time [3], almost all research to date on the ideology–environmentalism link has been cross-sectional in nature. It is therefore inappropriate to draw comments on the direction of any temporal association before more longitudinal research has been conducted.

Despite some positive evidence for the relationship between RWA and environmentalism, research that includes SDO tends to have the effect of RWA reduce or disappear. For example, Milfont et al. [10] showed that SDO accounts for unique variance in environmental attitudes over and above RWA, indicating again that SDO is the stronger predictor. Consistent with these findings, Häkkinen and Akrami [9] did not find evidence of a RWA–environmentalism link. Of the ideological variables included in their study, SDO was the strongest predictor of climate change denial, whilst RWA was not a significant predictor (Studies 1 and 2). However, both Schultz and Stone [12] and Peterson et al. [14] found evidence for the RWA–environmentalism link while examining environmental issues with RWA as the only ideological correlate. Taken together, these findings suggest that perhaps the RWA–environmentalism relationship is driven by the Authoritarian tie with SDO (the shared variance indicated by the weak correlation typically found between SDO and RWA [22]).

Synthesizing research to date that relates these ideological attitudes to environmentalism, Stanley and Wilson [23] demonstrated that SDO and RWA predict lower environmentalism both together and independently. They explored the possibility that the shared variance between SDO and RWA is driving the RWA–environmentalism link by meta-analysing regression coefficients to assess the independent contribution of each ideology in predicting environmentalism. They showed that, contrary to the pattern of results implied by limited empirical evidence presented here, each ideological attitude has a comparable unique association with environmentalism. However, they also show that the association between SDO and environmentalism is stronger among general population samples than when the same relationship is examined using student samples. This finding could explain the non-significant longitudinal path from SDO at time one to environmentalism at time two in Stanley et al.’s [15] study, which relied on a student sample.

Previous research relying on cross-sectional data therefore suggests that both SDO and RWA are key variables in the ideology–environmentalism link, but that the strengths of these associations depends on the sample type. As stated earlier, Stanley et al. [15] were the first to examine this relationship longitudinally, demonstrating that RWA predicted climate change denial most strongly over time, while SDO was not a significant predictor. As this research drew from a student sample, the results are therefore consistent with Stanley and Wilson’s [23] findings that SDO exhibits a weaker association with environment-relevant variables in these samples. The present study advances this research by examining the impact of SDO and RWA on environment-related attitudes in a general population sample, spanning five years.

### Current study

Our study specifically examines the influence of SDO and RWA on people’s willingness to change their behaviour to protect the environment in a New Zealand general population sample. We examine how these ideological variables predict environmentalism across five time points, expecting to find firstly that each variable evidences strong stability over time. This would be consistent with Stanley et al.’s [15] finding, where both ideological and environmental attitudes remain largely unchanged over time. We also expect the longitudinal association with environmentalism to be consistent with the majority of cross-sectional literature drawing from general population samples, and therefore show SDO to be a stronger predictor than RWA.

Previous studies linking these ideologies to lower environmentalism imply that individuals relatively high in SDO and RWA have more negative environmental attitudes *because* of these ideologies, hence suggesting a temporal relationship that might flow from ideology to environmentalism, which fits with theoretical models of how SDO and RWA relate to attitudes [24]. However, there is limited empirical evidence to suggest that environmentalism also predicts changes in SDO over time [15, 25]. This would mean that environmentalism drives ideology, which is somewhat consistent with literature showing that attitudes can be drivers of ideas [26], but inconsistent with Duckitt et al.’s [24] model, which implies a causal flow from ideology to attitudes. Drawing from both theory and this past research, we may find that environmentalism also predicts changes in ideology.

Duckitt [3] shows relatively strong cross-sectional associations between SDO and RWA in New Zealand, while in other contexts the relationship tends to be weaker or non-significant. He posits the strong SDO-RWA association might be due to the highly polarized nature of the political system in New Zealand. Duckitt suggests that RWA and SDO exhibit positive bidirectional associations over time, as cognitive consistency pushes endorsers of each to increase endorsement of the other ideology. Therefore, on the association between SDO and RWA over time, we expect reciprocal cross-time effects.

## Method

### Participants and procedure

We used data from the New Zealand Attitudes and Values Study (NZAVS) [27], a nationally representative, longitudinal study of personality, social attitudes, and health outcomes. The present study uses data from individuals who participated in the first five years of the NZAVS (from 2009 to 2013). Of the 22,966 participants included in this study, the average age was 43 years at Time 1 (2009; *M* = 43.07, *SD* = 14.67) and 62% were female. Furthermore, 76% of participants were born in New Zealand and the majority (86%) identified as Pākehā (New Zealand European). The University of Auckland Human Participants Ethics Committee approved all procedures, and participants gave informed written consent. Full details about the sample, including the procedure, retention, and demographics of participants, are available on the NZAVS website.

### Measures

The measures used in this study are included in S1 File.

*Social dominance orientation* was assessed using six items from Sidanius and Pratto’s [1] SDO_6_ scale. Three items favoured dominance, such as “To get ahead in life, it is sometimes okay to step on other groups”, and three favoured equality, such as “We should have increased social equality”. Participants rated the extent to which they agreed with each item on 7-point Likert scales, from 1 (strongly disagree) to 7 (strongly agree).

*Right-wing authoritarianism* was assessed with six items from Altemeyer’s [28] RWA scale. These included items such as “Our country will be destroyed someday if we do not smash the perversions eating away at our moral fibre and traditional beliefs”. The item “Some of the best people in our country are those who are challenging our government, criticizing religion, and ignoring the “normal way” things are supposed to be done”, was not assessed at time two, but included in all other time points. Again, responses were on a scale from 1 (strongly disagree) to 7 (strongly agree).

*Environmentalism* was measured as individual’s willingness to make sacrifices for the environment. These items were modelled from Liu and Sibley’s [29] two items on sacrifice intentions, “Are you willing to change your daily routine in order to protect the environment?” and “Are you willing to make sacrifices to your standard of living (e.g., accept higher prices, drive less, conserve energy) in order to protect the environment?”. Participants responded on a scale from 1 (definitely no) to 7 (definitely yes), with a midpoint of 4 (maybe).

### Data analysis

We used a cross-lagged structural equation model to infer causal associations between SDO, RWA and willingness to sacrifice for the environment. The model thus included three latent variables across five measurement points, where each variable within a time point was set to predict each variable in the subsequent time point. The autoregressive cross-lagged panel model allows examination of reciprocal influences among the three variables and was conducted in Mplus (version 7.3 [30]). A copy of the Mplus syntax used will be available on the NZAVS website upon acceptance of the manuscript. We used full information maximum likelihood (FIML) to impute missing data, as this has been shown to outperforms other approaches [31].

We tested the reciprocal influences between the constructs with a stationary cross-lagged model where all parameters are set as time-invariant. We started by constraining the factor loadings of our latent variables to equality so that, for each factor, the individual items that make up the factor were set to be equal across time points. For example, item 1 in the SDO scale was set to equality across all five time points, as was item 2, and so on for SDO and the other variables to ensure factor loadings were invariant across measurement points. Next, we constrained the means (intercepts) of the factors to equality. Put concretely, this made the latent means of the three constructs invariance from Time 1 to Time 5. We also constrained to equality the residual variance for the same item over time, so for example item 1 in the SDO scale had equal residual variance across all time points. Finally, we constrained the cross-lagged associations between the constructs to be the same in each measurement point. We imposed this stationary cross-lagged model because we were more interested in the overall and comparative effects of SDO and RWA on changes in environmentalism, and not on whether these effects change during the interval measured.

We present unstandardized parameters, which are invariant over time due to the constraints placed on the model. Because the standardized estimates are useful for comparing strengths of paths, we also include a range of beta weights alongside the unstandardized parameters. Due to the large sample size of this dataset, we adopted a conservative *p*-value threshold of *p* < .01 to indicate significance of findings (see also [32]).

## Results

Table 1 presents the descriptive statistics and correlations between our variables across all time points. Reliabilities for SDO and RWA were poorer than is typically the case when the full scales are used, but are consistent with expectations based on previous research using short measures [33]. Of note here is that the zero-order correlation between SDO and environmentalism was stronger than the correlation for RWA, as indicated by the non-overlapping confidence interval. Nonetheless, both ideological variables are negatively related to willingness to make sacrifices for the environment.

**Table 1:** Descriptive statistics and correlations between variables across all time points.

		**TIME 1**			**TIME 2**			**TIME 3**			**TIME 4**			**TIME 5**		
		SDO	RWA	WILL	SDO	RWA	WILL	SDO	RWA	WILL	SDO	RWA	WILL	SDO	RWA	WILL
**TIME 1**	SDO	-														
	RWA	.20 [.16, .24]	-													
	WILL	-.28 [-.32, -.23]	-.11 [-.16, -.07]	-												
**TIME 2**	SDO	.68 [.65, .70]	.21 [.16, .25]	-.24 [-.28, -.20]	-											
	RWA	.19 [.15, .22]	.79 [.77, .81]	-.08 [-.12, -.03]	.22 [.18, .26]	-										
	WILL	-.24 [-.28, -.19]	-.08 [-.13, -.03]	.63 [.59, .66]	-.25 [-.29, -.20]	-.05 [-.10, -.04]	-									
**TIME 3**	SDO	.65 [.62, .68]	.22 [.17, .26]	-.21 [-.26, -.17]	.69 [.66, .71]	.20 [.16, .24]	-.22 [-.27, -.17]	-								
	RWA	.20 [.16, .24]	.79 [.78, .81]	-.12 [-.16, -.08]	.23 [.19, .27]	.79 [.78, .81]	-.08 [-.13, -.04]	.25 [.21, .29]	-							
	WILL	-.25 [-.30, -.21]	-.11 [-.15, -.07]	.62 [.59, .65]	-.23 [-.28, -.19]	-.08 [-.12, -.03]	.67 [.63, .69]	-.24 [-.29, -.20]	-.11 [-.16, -.07]	-						
**TIME 4**	SDO	.63 [.59, .66]	.21 [.17, .26]	-.26 [-.30, -.21]	.68 [.66, .71]	.21 [.17, .25]	-.24 [-.29, -.20]	.68 [.65, .70]	.23 [.19, .28]	-.26 [-.30, -.21]	-					
	RWA	.20 [.15, .24]	.79 [.77, .81]	-.13 [-.17, -.08]	.22 [.18, .26]	.79 [.77, .80]	-.09 [-.14, -.04]	.23 [.18, .27]	.81 [.79, .82]	-.12 [-.16, -.07]	.26 [.22, .30]	-				
	WILL	-.26 [-.30, -.21]	-.10 [-.14, -.05]	.60 [.57, .63]	-.24 [-.28, -.20]	-.07 [-.12, -.03]	.63 [.60, .67]	-.27 [-.31, -.22]	-.13 [-.17, -.08]	.67 [.64, .70]	-.27 [-.31, -.23]	-.12 [-.17, -.08]	-			
**TIME 5**	SDO	.58 [.54, .61]	.18 [.13, .22]	-.28 [-.32, -.24]	.60 [.57, .63]	.16 [.12, .20]	-.26 [-.30, -.21]	.64 [.61, .67]	.19 [.15, .23]	-.28 [-.32, -.23]	.66 [.63, .68]	.21 [.17, .25]	-.31 [-.35, -.26]	-		
	RWA	.18 [.14, .23]	.77 [.75, .79]	-.12 [-.16, -.07]	.21 [.16, .25]	.76 [.75, .78]	-.09 [-.14, -.05]	.21 [.17, .25]	.79 [.78, .81]	-.12 [-.17, -.08]	.24 [.20, .28]	.81 [.79, .82]	-.15 [-.20, -.11]	.19 [.14, .23]	-	
	WILL	-.25 [-.29, -.21]	-.11 [-.15, -.06]	.56 [.52, .60]	-.23 [-.28, -.19]	-.08 [-.13, -.03]	.60 [.56, .63]	-.24 [-.28, -.19]	-.11 [-.15, -.06]	.65 [.62, .68]	-.25 [-.29, -.21]	-.13 [-.18, -.09]	.67 [.64, .69]	-.30 [-.34, -.26]	-.12 [-.17, -.08]	-
	α	.69 [.68, .70]	.70 [.69, .71]	.76 [.75, .77]	.70 [.69, .72]	.66 [.64, .68]	.77 [.75, .78]	.75 [.74, .76]	.73 [.71, .74]	.81 [.80, .82]	.73 [.72, .73]	.71 [.70, .72]	.79 [.78, .80]	.69 [.68, .69]	.69 [.69, .70]	.78 [.77, .78]
	Mean	2.54 [2.50, 2.58]	3.47 [3.42, 3.53]	4.93 [4.88, 4.99]	2.49 [2.45, 2.53]	3.36 [3.31, 3.41]	5.26 [5.21, 5.31]	2.46 [2.42, 2.50]	3.45 [3.40, 3.50]	5.11 [5.06, 5.16]	2.44 [2.40, 2.48]	3.40 [3.35, 3.45]	5.14 [5.08, 5.19]	2.38 [2.35, 2.42]	3.43 [3.39, 3.48]	5.19 [5.13, 5.24]
	SD	.95 [.92, .97]	1.16 [1.13, 1.19]	1.37 [1.33, 1.41]	.90 [.88, .93]	1.17 [1.13, 1.20]	1.19 [1.15, 1.23]	.90 [.87, .93]	1.11 [1.08, 1.14]	1.26 [1.21, 1.30]	.89 [.86, .91]	1.10 [1.07, 1.13]	1.24 [1.19, 1.28]	.84 [.81, .86]	1.10 [1.06, 1.13]	1.27 [1.23, 1.32]

*Note*. SDO = social dominance orientation, RWA = right-wing authoritarianism, WILL = willingness to make sacrifices for the environment. Numbers in square brackets represent 95% confidence intervals based on 1000 iterations. These values are based on participants who completed all items at all time points only (N = 2041).

Overall, model fit for the latent stationary cross-lagged model was poorer for those indices affected by model complexity (*χ*^2^(2279) = 36519.58, *p* < .001, CFI = .86), though given that our sample size was so large, it is very unlikely to obtain a non-significant chi-square. Those indicators less influenced by complexity pointed to good fit (RMSEA = .03, 90% CI [.025, .026], SRMR = .07), therefore the model was deemed acceptable.

### Stability over time: Autoregressive paths

**Fig 1:**
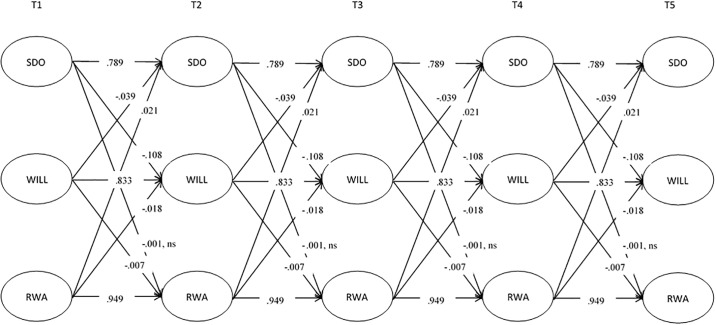
Unstandardized parameters assessing the association between SDO, RWA, and willingness to sacrifice for the environment (WILL). All meet our accepted threshold for significance of *p* < .01 unless otherwise noted.

**Fig 2:**
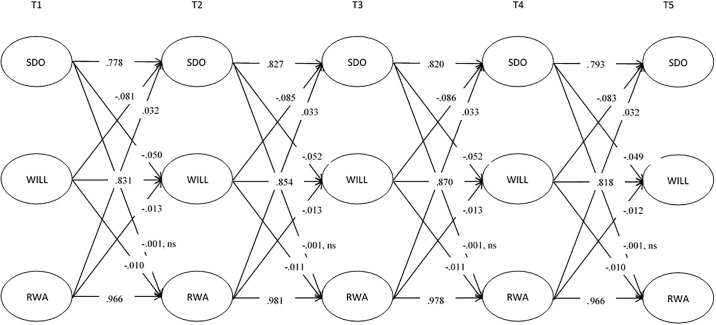
Standardized parameters assessing the association between SDO, RWA, and willingness to sacrifice for the environment (WILL). All meet our accepted threshold for significance of *p* < .01 unless otherwise noted.

As shown in Figs 1 and 2, across all time points, the autoregressive path for willingness to sacrifice was strong (B = .83, SD = .01, *p* < .001, 95% CI [.82, .84]; β’s range from .83-.87), indicating that an individuals’ level of willingness to make sacrifices for the environment is relatively stable over time. Consistent with our prediction that ideological variables remain largely unchanged over time, the autoregressive paths for both SDO (B = .79, SD = .01, *p* < .001, 95% CI [.78, .80]; β’s range from .78-.83) and RWA (B = .95, SD = .003, *p* < .001, 95% CI [.94, .96]; β’s range from .97-.98) were similarly stable over time.

### Ideology predicting environmentalism over time: Cross-lagged paths

The findings from our cross-lagged paths provide support for past research that has indicated SDO to be an important predictor of environment-related attitudes. Indeed, SDO was a stronger predictor of change in environmental sacrifice (B = -.11, SD = .01, *p* < .001, 95% CI [-.13, -.09]; all β’s -.05) than RWA (B = -.02, SD = .01, *p* = .001, 95% CI [-.03, -.01]; all β’s -.01). Due to the nature of the analysis and the constraints placed on the model, the traditional Wald test to confirm that SDO is a statistically stronger predictor than RWA is not possible. Instead, we imposed similar constraints on the model as in the Wald test: we constrained the paths to equality from SDO to environmentalism and from RWA to environmentalism. We then compared the fit of this model that assumes no difference in the predictive ability of these variables to our main model, where the paths are not constrained to equality. We found that our original model provides significantly better fit to the data Δ*χ*^2^(1) = 51.86, *p* < .001. Comparing the strengths of the standardized paths, we conclude that SDO is a statistically stronger predictor of change in willingness to sacrifice for the environment than RWA in our sample.

The lagged effect of willingness to sacrifice on both SDO and RWA were statistically significant and negative (B = -.04, SD = .002, *p* < .001, 95% CI [-.04, -.03]; β’s between -.08 and -.09 and B = -.01, SD = .002, *p* = .002, 95% CI [-.01, -.003]; all β’s -.01, respectively). These results indicate a bidirectional association between ideology and environmentalism over time: SDO and RWA predict change in willingness to sacrifice for the environment, and vice versa. However, while the association between SDO and environmentalism was bidirectional, the lagged effect of environmentalism on SDO was stronger than the reverse (Δ*χ*^2^(1) = 40.76, *p* < .001), whilst there was no difference between the strength of the effect of RWA on environmentalism and vice versa (Δ*χ*^2^(1) = 2.58, *p* = .108). These analyses help us disentangle the directionality of the relationships, and suggest that, contrary to our hypothesis, environmental attitudes have a stronger influence on SDO over time than the reverse.

Finally, we examined the bidirectional association between SDO and RWA. Whilst the lagged effect of RWA on SDO was statistically significant (B = .02, SD = .003, *p* < .001, 95% CI [.02, .03]; all β’s .03), SDO did not predict change in RWA (B = -.001, SD = .01, *p* = .844, 95% CI [-.01, .01]; β’s -.001).

## Discussion

We assessed how SDO, RWA, and environmentalism are related over time in a general population sample. Consistent with our predictions and the majority of cross-sectional research, across five years SDO was the stronger ideological predictor of environmentalism, while RWA exhibited a weaker cross-time association. However, environmentalism also significantly related to changes in ideology over time, and while there was no difference in the strength of the path from RWA to environmentalism and the reverse, contrary to our hypothesis, we found that *environmentalism* is more strongly related to changes in SDO than the reverse. These results suggest that there is a more likely potential causal pathway from environmentalism to ideological attitudes than the opposite direction. We elaborate on each main finding in the next sections.

### Environmental attitudes predict ideological attitudes over time

We show that environmentalism predicts ideology over time. This replicates earlier findings by Stanley et al., [25] who showed that the two dimensions of SDO, anti-egalitarianism and dominance, differentially predicted environmentalism cross-sectionally, while longitudinally it is *environmental attitudes* that predict the components of SDO over time (and not vice versa). This led to the conclusion that we ought to be agnostic about whether ideology precedes environmental attitudes, or the other way around, as further research was needed (see Stanley et al. for a full discussion).

While there is little research on the temporal order and inter-relationships of SDO, RWA and environmentalism specifically, in the sphere of intergroup relations Duckitt’s [3] dual-process model predicts a pathway where ultimately SDO and RWA are causes of prejudiced attitudes. Our study contributes to the literature by demonstrating that *both* directions are significant, and hence that ideological attitudes and environmental attitudes act on each other over time, though interestingly, environmentalism acts on SDO more strongly than the reverse.

The refusal to make personal sacrifices for the environment might lead individuals towards preferring an unequal society. Inaction on climate change means accepting its consequences and allowing these to fall to low status groups. While climate change is arguably the biggest environmental problem of our time, people tend to view themselves and those close to them as less vulnerable to its effects. This is because people are inclined to view climate change as psychologically distant, with themselves and people like themselves more immune to the effects, which are imagined as instead affecting distant social groups, locations, generations, or being uncertain [34–35].

There is some truth to these perceptions: Although humans are causing the rapid heating of the planet, the key contributors to climate change and those most vulnerable to its effects are not necessarily the same people. The IPCC [36] note that hazards associated with climate change are likely to exacerbate stressors and consequently effect people’s livelihoods, but these effects are not unilateral. Instead, the poor will be most affected, and they are also the least able to afford to adapt to the effects of climate change. Those in developing countries with low incomes are also most likely to experience negative consequences of climate change on their health. As well as those in poorer nations being most vulnerable to climate change overall, disadvantaged groups within these and other countries tend to be most at risk, thus prompting the IPCC [36] to warn that climate change might increase social inequality.

The issue of climate change therefore fits within the wider discussion about environmental justice [7, 8, 37–40]. Bryant [41] noted that environmental racism occurs when people of colour are disproportionately exposed to environmental hazards, and receive less protection from these hazards, than more privileged groups. Pellow [42] takes this idea a step further in defining environmental inequality as more strongly tied to social hierarchy, and the burdening of low-status social groups with environmental hazards (broadly defined). Indeed, individuals in subjugated groups tend to be exposed to more hazards in their jobs, and live in areas closer to landfills, pollution, and other environmental hazards. Pellow argues that this inequality occurs when competition for resources ultimately results in dominant groups gaining disproportionate access to these resources, and low-status or minority groups having to instead face the consequences of environmental degradation.

Despite this state of affairs being unfair, some individuals prefer it this way. Specifically, individuals who endorse social dominance orientation are accepting and even *prefer* social inequality and hierarchical intergroup relations [1]. They believe that high-status social groups are at the top of the hierarchy because they are better than other, subjugated groups. As such, they believe they deserve to disproportionately benefit from environmental exploitation and avoid environmental hazards, while disregarding the effect this has on lower-status social groups. Indeed, Social Dominants are accepting of environmental degradation when the benefits go to high-status social groups, and when given the choice prefer to direct environmental hazards into areas occupied by members of low-status groups [5–6].

With our findings of a temporal connection between environmentalism and SDO, this clarifies the potentially causal nature of this association by suggesting that environmental attitudes precede—and are perhaps foundational for—hierarchical attitudes. It is possible that rejecting environmental action, when doing so is likely to exacerbate problems for low status groups, evokes feelings of cognitive dissonance. These are resolved through accepting social hierarchy and inequality as legitimate and fair (i.e., holding a social dominance orientation). Therefore, individuals who tolerate the environmental inequality brought on by the current environmental crisis, by way of forgoing making personal sacrifices to mitigate the problem, are more likely to extend these attitudes to general tolerance for social inequality.

Jylhä and colleagues [7, 8, 40] advanced the idea that individuals who endorse SDO are more likely to reject the reality of climate change as they are also more tolerant of the inherent inequity in both the causes and consequences of climate change. Denial may therefore serve as a way of maintaining or enhancing the status difference between social groups [8, 10]. Our research runs parallel to this interpretation, by suggesting that SDO may serve as a way of protecting the individual from discomfort brought about by the social consequences of inaction on climate change. Sidanius and Pratto’s [1] theory of social dominance states that in each society, a hierarchy exists where membership in a high-status group comes with more privileges than membership in a low-status group. Those endorsing SDO are more accepting of ideologies such as racism and sexism, which act to strengthen the hierarchy, and oppose multiculturalism and feminist beliefs, which attenuate group-based dominance. Jost and Hunyady [43] noted that inaction on intergroup inequality by low-status group members likely elicits dissonance, and this is alleviated by accepting the social system as legitimate. Gaining similar opposition from Social Dominants, environmentalism is a hierarchy-attenuating ideology: Exploitation of natural resources favours the dominant group, while the consequences fall to those at the bottom of the hierarchy. Whether directed towards other humans, animals, or humans through the use of the environment, prejudice ultimately reveals Social Dominant’s motivation for power in what they perceive as a competitive world [3, 44], or perhaps reliance on this conservative political ideology in response to the threat of change [45].

An alternative explanation for this temporal association is based on the measure of environmentalism we use in this study: willingness to make sacrifices for the environment. Duckitt’s [3, 24] work suggests holding an SDO stems from the belief that individuals must compete for resources. Reluctance to reduce one’s own access to resources (through making sacrifices for the environment) is consistent with a competitive worldview, hence potentially explaining why endorsement of this variable precedes changes in SDO. More research is needed to further explore the potential mechanisms underlying the association.

### Ideological attitudes predict environmental attitudes over time

Our pattern of results revealed a set of bidirectional ideology-environmentalism associations. Furthermore, of the two ideological predictors included in this study, SDO is a stronger predictor of changes in environmentalism over time than RWA. This is the reverse of the pattern obtained from the first longitudinal analysis on this topic, which showed that RWA was the stronger predictor among students [15]. However, this difference in findings is consistent with expectations based on the results of Stanley and Wilson’s [23] meta-analysis, which showed that the association with SDO is significantly weaker in student samples. It is likely that the difference in sample type therefore accounts for the differences in our results, as we used a general population sample. In this context, *both* SDO and RWA are related to environmentalism, but SDO is a significantly stronger prospective predictor than RWA.

For those in the general population, it is their beliefs about how social groups should be organized that inform their environmental attitudes to a greater degree than their attitudes about authority. However, the bidirectionality of our findings means they warrant exploration of alternative explanations for what drives the associations. For instance, the relationship between ideology and environmentalism across time could be explained by a third variable. Specifically, it is possible that something related both to ideological and environmental attitudes could drive changes in each variable independently, hence explaining the apparent causal relations. For instance, economic factors are related to intergroup attitudes, with some evidence that resource scarcity and economic recession increases intergroup conflict and discrimination [46–47]. These situational factors could also reasonably motivate self-serving behaviours, such as forgoing making personal sacrifices for the environment. Future research could test these and other alternative explanations by examining whether the ideology–environmentalism link is explained by perceptions of resource scarcity or economic downturn.

Our findings also warrant caution for researchers wanting to increase pro-environmentalism: targeting endorsers of SDO with climate change communications might be futile. Specifically, if endorsement of SDO does little to influence later levels of environmentalism (at least, less than the reverse direction), it is unclear whether and how interventions might succeed in spurring high-SDOs to action. In one recent study, Zhao and colleagues [48] showed that an intervention that reduced endorsement of SDO increased environmentalism. Future research ought to continue exploring the potentially causal nature of the ideology–environmentalism association. In the case of environmental action, this research also offers hope that if environmental action can be achieved and sustained, this might flow through to reducing endorsement of SDO and its correlates (e.g., prejudice [3]).

### Strengths and limitations

Our research uses a nationally representative sample, meaning that our results are highly generalisable, at least to the New Zealand population. We have also replicated the initially surprising finding of a stronger temporal association between environmentalism and SDO [15], which increases our confidence in this directional relationship. Despite this, there are some limitations of our study to address. As our research was survey-based, and participants’ responses were restricted to Likert-type responses to question items. Although this is a sufficient way to test people’s attitudes and results from the autoregressive paths indicate the scores are stable over a five-year interval, it is unclear *why* participants chose the response options that they chose. Specifically, from our results we cannot pinpoint the rationale behind why those less willing to behave pro-environmentally score higher in SDO. Future research could expand on our knowledge of the ideology–environmentalism link by using qualitative methods, such as an interview study, that probes the rationale behind people’s attitudes on the environment. Exploiting the environment might be less about dominance over nature, and more about dominance over others *by way of* the environment (as in [5]). This is an important distinction, given that the motivation underlying Social Dominant’s opposition to environmental action might affect how they interpret information about climate change.

## Conclusion

Using a nationally representative sample, we tested the strength of associations between SDO, RWA, and environmentalism over time. We found that both SDO and RWA predict lower willingness to make sacrifices for the environment over time, but SDO is the stronger predictor. Furthermore, the direction of the relationship is more strongly from environmentalism to SDO than the reverse. This result indicates that pro-environmentalism is associated with a rejection of the desire to dominate in the future. We interpret this to mean that individual inaction on environmental issues is incompatible with striving for social equality.

## Supporting information

S1 File Stimulus materials. (DOCX)
