# Supplementary material for: The influence of social dominance orientation and right-wing authoritarianism on environmentalism: A five-year cross-lagged analysis
Source: PLoS One. 2019 Jul 10;14(7):e0219067. doi: 10.1371/journal.pone.0219067 (PMC6619689; doi:10.1371/journal.pone.0219067)
Supplement: S1 File — (DOCX) [file pone.0219067.s001.docx]

**Stimulus materials**

Social Dominance Orientation items included at all time points

1. It is okay if some groups have more of a chance in life than others
2. Inferior groups should stay in their place
3. To get ahead in life, it is sometimes okay to step on other groups
4. We should have increased social equality
5. It would be good if groups could be equal
6. We should do what we can to equalize conditions for different groups

Right Wing Authoritarianism items included at all time points

1. It is always better to trust the judgment of proper authorities in government and religion than to listen to the noisy rabble-rousers in our society who are trying to create doubt in people’s minds
2. It would be best for everyone if the proper authorities censored magazines so that people could not get their hands on trashy and disgusting material
3. Our country will be destroyed someday if we do not smash the perversions eating away at our moral fibre and traditional beliefs
4. People should pay less attention to the Bible and other old traditional forms of religious guidance and instead develop their own personal standards of what is moral and immoral
5. Atheists and others who have rebelled against established religions are no doubt every bit as good and virtuous as those who attend church regularly

Excluded item (missing from time 2)

1. Some of the best people in our country are those who are challenging our government, criticizing religion, and ignoring the “normal way” things are supposed to be done

Environmental Sacrifice

- 1. Are you willing to make sacrifices to your standard of living (e.g., accept higher prices, drive less, conserve energy) in order to protect the environment?
  2. Are you willing to change your daily routine in order to protect the environment?
